# Application of ischemic postconditioning's algorithms in tissues protection: response to methodological gaps in preclinical and clinical studies

**DOI:** 10.1111/jcmm.13159

**Published:** 2017-04-12

**Authors:** Saeid Feyzizadeh, Reza Badalzadeh

**Affiliations:** ^1^ Drug Applied Research Center Tabriz University of Medical Sciences Tabriz Iran; ^2^ Cardiovascular Research Center Tabriz University of Medical Sciences Tabriz Iran

**Keywords:** ischaemic postconditioning, cardioprotection, ischaemia–reperfusion injuries, clinical studies, methodology

## Abstract

Ischaemic postconditioning (IPostC) was introduced for the first time by Zhao *et al*. as a feasible method for reduction of myocardial ischaemia–reperfusion (IR) injury. The cardioprotection by this protocol has been extensively evaluated in various species. Then, further research revealed that IPostC is a safe and convenient approach in limiting IR injury of non‐myocardial tissues such as lung, liver, kidney, intestine, skeletal muscle, brain and spinal cord. IPostC has been conducted with different algorithms, resulting in diverse effects. The possible important factors leading to these differences are the difference in activation levels of signalling pathways and protective mediators by any algorithm, presence or absence of IPostC effectors in each tissue, or intrinsic characteristics of the tissues as well as the methodological biases. Also, the conflicting results have been shown with the application of the same algorithm of IPostC in certain tissues or animal species. The effectiveness of IPostC may depend upon various parameters including the species and the tissues characteristics. For example, different heart rates and metabolic rates of the species and unequal amounts of perfusion and blood flow of the tissues should be considered as the important determinants of IPostC effectiveness and should be thought about in designing IPostC algorithms for future studies. Due to these discrepancies, there is still no optimal single IPostC algorithm applicable to any tissue or any species. This issue is the main topic of the present article.

## Introduction

The concept of reducing reperfusion injury in the animal models of myocardial infarction (MI) and in patients undergoing surgery for the opening of coronary artery stenosis was introduced for the first time in the late 1970s by Buckberg *et al*. 1, 2. Myocardial reperfusion injury leads to a series of complications including coronary vasculature dysfunction, activation of endothelium‐derived adhesive molecules and migration of inflammatory cells to the damage area, tissue oedema, electrophysiological dysfunctions, myocardial infarction and apoptosis 3. Therefore, finding the new strategies to reduce the complications of the process and improve the patients’ outcome is clinically very important 4. In 2003, Zhao *et al*. were the first researchers to use the term ischaemic postconditioning (IPostC) as a potential approach to increase the resistance of the heart against myocardial damages induced by ischaemia and reperfusion (IR) injury 5, 6. IPostC is performed in the form of a series of repeated cycles of reperfusion/ischaemia with the specific timings and episodes, applied immediately at the onset of reperfusion. The studies showed that this strategy leads to a significant reduction in ventricular arrhythmias and considerably reduces the infarct size of the ischaemic heart 7, 8. Although it does not seem that cardioprotective effect of IPostC is stronger than those of ischaemic preconditioning (which is applied before the index ischaemia), the conclusions of investigations from different laboratories indicate its protective potency against IR insults not only in the heart but also in different tissues of many species 7, 9. However, despite extensive pre‐clinical studies indicating the positive effects of IPostC especially in the field of cardioprotection, the translation of IPostC results from animal studies to the clinical setting has been encountered with problems. Some clinical studies using IPostC protocols have reported positive effects 10, 11, 12, while others have showed no or negative results 13, 14. On the other hand, many research with considerable results has been carried out in the field of protective effects of IPostC in different tissues of animal models 15, 16, 17, 18, 19, 20, 21, 22, but in humans, in spite of notable research on myocardial IR injury, there is a few or lack of study in the field of the effectiveness of IPostC strategy on the protection of other human tissues such as kidney, liver, lung, intestine, and brain 23. In the case of renal protection, there is only one pilot study (with 20 patients undergoing kidney transplantation), in which IPostC strategy (3 × 1 min.) could not exert considerable effects on renal delayed graft function 3 months after transplantation 24. There was no significant difference in reducing effects of IPostC on serum creatinine levels between experimental and corresponding control groups 24. In addition, in a study conducted by Ricca *et al*. on 50 patients undergoing liver transplantation, the effects of IPostC on AST levels and other markers of liver function were similar with those of control patients. Even in some patients in this study, the histological damage was greater in IPostC‐receiving group, and this protocol could not reduce the apoptosis but instead increased significantly the level of autophagy in periportal areas 25. There is no study in the case of other tissues in human. Therefore, as human studies in the field of IPostC effects on the protection of non‐cardiac organs are very limited, it is too early to achieve a definite conclusion regarding the efficacy of this strategy in protecting these tissues in humans. Although the primary scope of this article is discussion about the reasons for the variation in the efficacy of IPostC algorithm in various tissues, the main reasons for the lack of translation of cardioprotective effects of IPostC from basic into clinical settings are discussed first.

## Optimization of IPostC‐induced cardioprotection indicators based on the translatable results from animal models to humans

In general, the main parameter involved in the lack of good translation of postconditioning (and other protective interventions) results from animal studies to the clinical setting is related to the issues about the designing of the basic and pre‐clinical studies, for example, inappropriate myocardial infarction models, and lack of attention to various pathophysiology of the ischaemia/reperfusion disorders, resulting in the implementation of a poorly designed study. In addition, most of the clinical studies do not appropriately think through the pre‐clinical data that has already been carried out, in their trials settings 26, 27. Many factors should be considered in designing the pre‐clinical and clinical studies to obtain a reliable and translatable result and reduce the burden of IR injury, which they are introduced below in the case of the cardioprotective efficacy of IPostC.

### Confounding factors in cardioprotective efficacy of IPostC

In patients with coronary heart disease, confounding factors could potentially alter the vulnerability of IR injury and negate the cardioprotective effects of clinical interventions. The most important confounder in human patients with myocardial IR injury is the presence of cardiovascular comorbidities and risk factors such as ageing, hypertension, hyperlipidaemia, atherosclerosis, diabetes mellitus, left ventricular hypertrophy and heart failure 28, 29, 30. These comorbidities can explain the weakness of cardioprotective effects of IPostC in the clinical setting and the majorities of pre‐clinical basic studies ignore the presence of them in designing their studies. These concomitant diseases, as well as drugs used for their treatments, may lead to critical changes in the activity of the molecular signalling pathways (see below) necessary for the cytoprotective efficacy of IPostC and myocardial salvage during IR insults 28.

In addition, most tissues in healthy individuals have an innate collateral blood flow that makes the ischaemic tissue to adapt the damaged conditions 31. Some studies have shown that above‐mentioned risk factors considerably impair the responsiveness of collateral flow in ischaemic conditions 32, 33. Thus, the inefficiency of IPostC strategies in clinical myocardial IR trials is also attributable to the insufficient coronary collateral circulation in the presence of comorbidities. Furthermore, in the case of percutaneous coronary interventions (PCI), repetitive inflation/deflation of angioplasty balloon as the cycles of IPostC at the beginning of reperfusion in atherosclerotic obstructed vessels may detach the sclerotic debris or endothelial cells as a result of balloon's expansion pressure, which they can block again the microvascular system, ultimately leading to the failure of coronary collateral circulation.

Another important confounding factor for cardioprotection by IPostC in patients is concomitant medication for controlling the cardiovascular comorbidities or timely treatment of acute myocardial infarction. These medications *per se* have shown their both inhibitory and activatory effects on cardioprotection in pre‐clinical and clinical studies 27, 30. Thus, both aspects of this result should be considered in interpretation and evaluation of the degree of IPostC success in cardioprotection. Inhibitory effects of concomitant medication can be one of the causes of the inefficiency of IPostC, and on the other hand, protective effects of these medications may lead to the manifestation of the false positive results by IPostC administration. Therefore, due to the mentioned interferences in cardioprotection, larger scale cohort studies should be conducted based on the presence of cardiovascular risk factors in certain subgroups to analyse more clearly the influences of age, gender, comorbidities and co‐treatments on IPostC protective efficacy 26.

### Lack of an optimized and standard algorithm in clinical trials

The IPostC has been applied with different algorithms in different clinical studies, for example, with three or four cycles of 30 or 60 sec. of repetitive reperfusion/ischaemia 34, 35. The lack of standard and optimized algorithm will lead to different results in various clinical studies. Comparison of the effectiveness of some similar IPostC algorithms in clinical studies is seen in Table 1. The same algorithm of IPostC has shown diverse results that may be related to the interference of confounders, discussed above.

**Table 1:** Comparison of the effectiveness of similar IPostC algorithms in clinical studies. As seen in the table, the same algorithm of IPostC has shown diverse results that may be related to the interference of confounders discussed in the text. The negative results of IPostC are usually seen when the duration of ischaemia is longer

Positive results			Neutral or negative results		
Study	Time of ischaemia	Zone of obstruction	Study	Time of ischaemia	Zone of obstruction
**IPostC Protocol**					
4 × 1 min. (inflations and deflations of angioplasty balloon)					
Staat *et al*. 34	≥6 hrs	Upstream of stent	Sorensson *et al*. 53	≥6 hrs	Within the stent (at the site of lesion)
Thibault *et al*. 52	≥6 hrs		Tarantini *et al*. 54	≤6 hrs	
Araszkiewicz *et al*. 12	≥6 hrs		Freixa *et al*. 55	≤12 hrs	
			Hahn *et al*. 56	≤12 hrs	
			Eitel *et al*. 35	…..	
4 × 0.5 min. (inflations and deflations of angioplasty balloon)					
Lonborg *et al*. 11	≤12 hrs	Within the stent	Dwyer *et al*. 14	≤6 hrs	Within the stent (at the site of lesion)
			DANAMI 3 13	≤12 hrs	

IPostC: ischaemic postconditioning.

### Long *versus* short durations of previous ischaemia

The time length of acute myocardial ischaemia is one of the main and primary determinants of the development of infarct size (Table 1). In comparison, this acute ischaemic time in patients with ST‐elevation MI (STEMI) was up to 12 hrs 36, while in animal MI modelling was usually lower than 3 hrs 37. This variation can significantly lead to the variances in IPostC‐induced cardioprotection among the basic and clinical studies. In addition, coronary artery obstruction in clinical cases occurs gradually and over the time and it varies from patient to patient, for example, it takes 5 months in one patient, but more than 2 years in another. Hence, the patients who have experienced this process will have different previous ischaemia experiences (in terms of time and intensity) before hospitalization. This process results in the induction of different intensities of preconditioning effects on ischaemic tissue, leading to different responsiveness of tissue to the IPostC protocols.

### The absence of a common standard method to assess the infarct size both in animal models and human patients

Different techniques such as cardiac biochemical markers, echocardiography, magnetic resonance imaging (MRI), computed tomography (CT), single‐photon emission computed tomography (SPECT) and positron emission tomography (PET) imaging, histology, tetrazolium chloride and propidium iodide staining are available to estimate the area at risk and infarct size 38, 39. Two standard methods SPECT and cardiovascular MRI are usually used for clinical cases 40, while in animal models, the staining methods are still used to assess the efficacy of cardioprotective strategies mainly due to the low cost of the method 39. Therefore, there is no common standard method, yet, in pre‐clinical and clinical studies to evaluate the infarct size as the main indicator of cardiac injury and protection. These different techniques for the estimation of IPostC outcome can lead to dissimilar, non‐comparable results.

### Variability of ischaemia region in the patients’ hearts because of the possibility of occlusion development along the coronary artery

**Figure 1:**
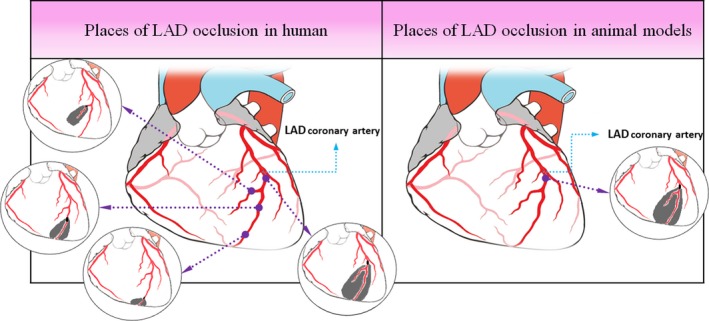
Graphic scheme of variations of ischaemic region in human and animal models of IR hearts. LAD, left anterior descending.

As seen in Figure 1, the regions for coronary occlusion in cardiac patients may vary throughout the entire length of the left anterior descending (LAD) coronary artery (any point from beginning to the end of the artery), while in animal modelling the ligation place is chosen by the researchers in a certain restricted place of LAD. So, displacement of LAD ligations and occlusions along the coronary artery would alter the severity of IR injury (the closer of occlusion to the origin of the LAD, more severe the IR extent, and vice versa), and this issue can influence the exact efficacy of cardioprotection.

### Previous history of experiencing mild ischaemia or receiving protective interventions

The other important factor that may impede the IPostC effects and should be considered in designing the clinical trials is the past history of experiencing mild ischaemia or receiving protective interventions like thrombolytic therapy and PCI in patients. Patients who have previously received one of such interventions, their hearts have been preconditioned yet and more likely IPostC would not be able to induce further protection.

### Time of IPostC administration in clinical case

As the body is influenced by daytime physiological changes such as alterations in hormonal levels, nervous system activity, body temperature and circadian rhythms (sleep and arousal), these changes may be also involved, even to some extent, in the IPostC intervention's efficacy. Thus, administration of IPostC intervention to patients during certain times of 24 hrs a day (morning, noon, evening or night) may lead to an even slight difference in the effectiveness of IPostC.

## Cellular mechanisms of IPostC effects

**Figure 2:**
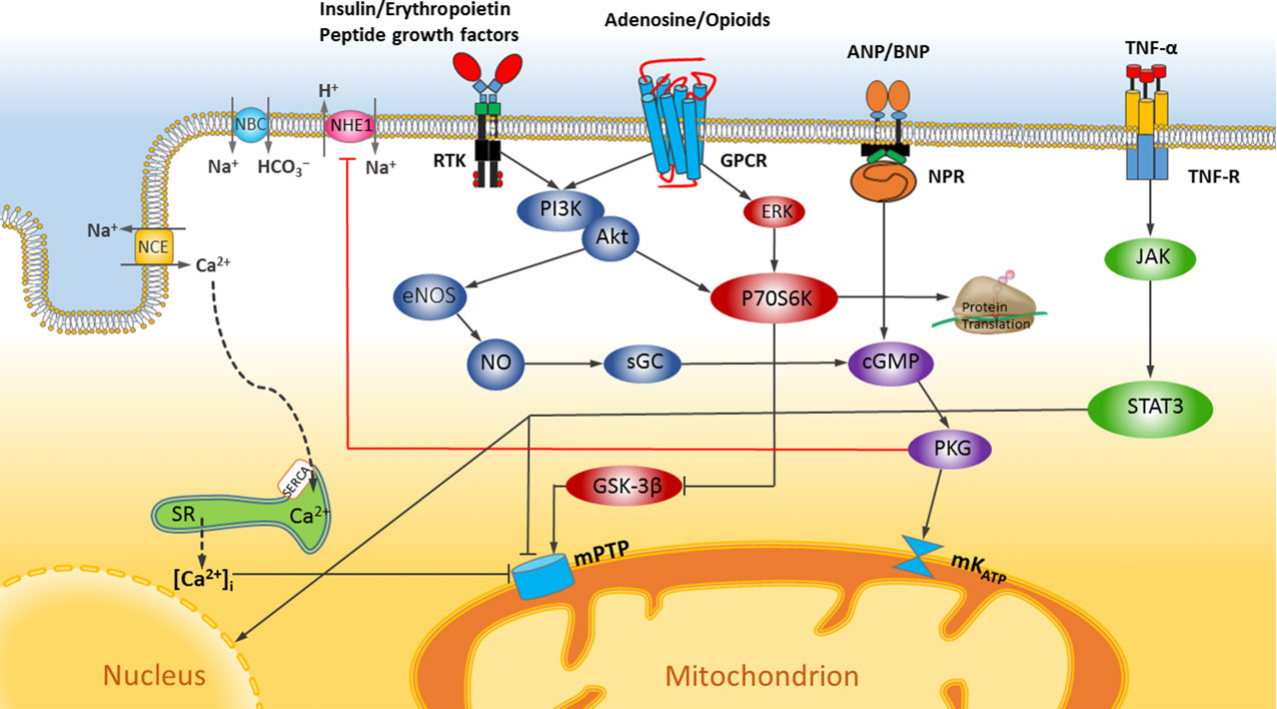
Schematic design for signalling pathways and mechanisms of IPostC in the myocardium. ANP, atrial natriuretic peptide; BNP, brain natriuretic peptide; NPR, natriuretic peptide receptor; PKG, cGMP‐dependent protein kinase; SERCA, sarcoplasmic/endoplasmic reticulum calcium ATPase; SR, sarcoplasmic reticulum; GSK‐3β, glycogen synthase kinase‐3β; mK_ATP_, mitochondrial K_ATP_ channel; mPTP, mitochondrial permeability transition pore; ROS, reactive oxygen species; NBC, Na^+^ bicarbonate co‐transporter; NCE, Na^+^/Ca^2+^ exchanger; NHE1, Na^+^/H^+^ exchanger; NO, nitric oxide; STAT3, signal transducer and activator of transcription‐3; JAK, Janus kinase; TNF‐α, tumour necrosis factor alpha; TNFR, TNF receptor; GPCR, G‐protein‐coupled receptors; eNOS, endothelial nitric oxide synthase; ERK, extracellular‐regulated kinase; RTK, receptor tyrosine kinase; PI3K, phosphatidylinositol‐3‐kinase; Akt, a serine/threonine kinase also known as protein kinase B; P70S6K, ribosomal protein S6 kinase beta‐1; sGC, soluble guanylyl cyclase.

The proposed mechanisms of actions of IPostC in the literature indicate that the protective effect of IPostC is essentially associated with reversing the reperfusion‐induced pathologies including oxidative stress, intracellular calcium overload, endothelial dysfunction and inflammatory responses 4. The most crucial finding is that the signalling pathways activated by IPostC during the initial minutes of reperfusion ultimately lead to the blockade of the mitochondrial permeability transition pores (mPTP), which its opening is a critical event leading to the cell death during reperfusion 41. Three separate signalling pathways for IPostC actions have been proposed: (1) reperfusion injury salvage kinase (RISK) pathway that includes the activation of PI3K/Akt and ERK and eventually inhibition of GSK‐3β; (2) survivor activating factor enhancement (SAFE) pathway that JAK/STAT and TNF‐α are the main components of this pathway; and (3) cGMP/PKG as a supplementary pathway, in which activation of natriuretic peptides and soluble guanylyl cyclase leading to the opening of mitochondrial ATP‐sensitive potassium channels. Although these pathways have been investigated separately, their cytoprotective activities eventually convergence on the mitochondria and especially inhibit the opening of mPTP. To better understand the signalling pathways of IPostC in the myocardium, see Figure 2.

Most pre‐clinical and some clinical studies have confirmed the necessity of RISK, SAEF and guanylyl cyclase signalling pathways for the effectiveness of cardioprotective interventions in IR injury. As the activation of different intracellular signalling pathways is based on type of intervention modalities and stimulation of cell membrane receptors, the presence and activity levels of these receptors on cells membrane and signalling machinery inside the cells of different tissues can determine the similarities or differences of mechanisms of protection in those tissues. Interestingly, the involvement of any of these signalling pathways in IPostC protection has been confirmed in certain species, but not in the others. For example, RISK pathway is the main mediator of the IPostC effects in the hearts of rats, while it is not the case in the guinea pig. In addition, the activation of this pathway by IPostC has been also shown in atrial muscle sampled from patients undergoing coronary artery bypass grafting (CABG) surgery. Accordingly, the studies have revealed that adenosine receptors (especially AR1 and AR3) show their protective roles through almost similar mechanisms in IR injury of various tissues such as heart, lung, kidney, liver, and brain 42, 43. In addition, Wen *et al*. 44 have indicated that the IPostC reduces IR injury in the small intestine through the activation of JAK/STAT pathway, however, Guo *et al*. 45 have reported that the effects of IPostC are achieved *via* Akt‐eNOS‐NO‐HIF signalling pathway in the hepatic IR insult. On the other hand, based on the studies of Chen *et al*. 46 and Zhang *et al*. 47, the RISK pathway plays a pivotal role in IPostC‐induced protection against IR injury in renal tissue. However, the reasons for these differences are unknown and it is still impossible to conclude with the certainty that whether different or similar signalling pathways mediate the protective effects of IPostC in different tissues.

## IPostC protocols in various tissues and its algorithms

Many experimental and clinical studies have shown that IPostC is a simple and safe approach to increase protection against damages caused by IR insults to various organs such as the heart, brain, kidney, liver, intestine, lung, skeletal muscle and spinal cord 9, 48. Despite the effectiveness of this method in various organs of humans and experimental models, many fundamental questions linked to the protective effects of IPostC are still unanswered. One of the main issues regarding the IPostC is its algorithm, that is, the number of each IPostC episode, and the duration or timing of each episode. In several tissues of different species, the IPostC is applied with different algorithms or protocols, for example, with three cycles of 30‐sec. reperfusion/30‐sec. ischaemia, six cycles of 10‐sec. reperfusion/10‐sec. ischaemia, or three cycles of 1‐min. reperfusion/1‐min. ischaemia. Although different algorithms of IPostC may put forth different effects in a certain tissue, the same algorithm has not similar effects in different tissues. Table 2 has summarized the IPostC algorithm of three cycles of 30‐sec. reperfusion/30‐sec. ischaemia used in human and animal studies with different tissues.

**Table 2:** Comparison of the effects of IPostC with the same algorithm of three cycles of 30‐sec. reperfusion and 30‐sec. ischaemia (30s/30s) in different organs undergoing ischaemia/reperfusion injury

Study	Species	Organ	I‐R protocol [IPostC algorithm: 3 (30sR/30sI)]	Main finding^a^	IPostC mechanisms
Luo *et al*. 57	Human	Heart	(Ischaemia with 56‐min. aortic cross‐clamping)‐(48‐hrs reperfusion follow‐up)	Reduction in CK‐MB but not TnI	IPostC also resulted in a reduction in inotrope requirement
Ma *et al*. 58	Human	Heart	(Revascularization after ≥12 hrs of AMI onset)‐(48‐hrs reperfusion follow‐up)	Improved WMSI, endothelial function, less CK, MDA	IPostC *via* enhancing CTFC, improve WMSI and decreased CK‐MB salvaging the coronary endothelial function and cardiomyocyte
Yang *et al*. 59	Human	Heart	(Revascularization after primary AMI)‐(7‐days reperfusion follow‐up)	27% reduced 72 hrs CK, 27% reduced MI SPECT 1 week, LVEF 44‐54%	IPostC following PCI significantly protects the heart against ischaemia/reperfusion‐induced injury
Fan *et al*. 60	Human	Heart	(Revascularization with primary PCI)‐(7‐days reperfusion follow‐up)	Reduction in peroxynitrite ‐induced nitro‐oxidative stress	The iNOS pathway a major mechanism whereby IPostC confers cardioprotection *in vivo*
Wagner *et al*. 51	Rat	Heart	30 min.–30 min.	28% reduction in myocardial infarct size *versus* control group	Reduced phosphorylation of GSK‐3β
Wagner *et al*. 51	Rat	Heart	20 min.–20 min.	60% reduction in myocardial infarct size	Reduced phosphorylation of GSK‐3β
Zhang *et al*. 61	Rat	Heart	30 min.–120 min.	50% reduction in myocardial infarct size	Improved oxidative status, and activities of Na/K‐ATPase, and Ca/Mg‐ATPase
Badalzadeh *et al*. 15	Rat	Heart	30 min.–120 min.	33% reduction in myocardial infarct size	Increased nitric oxide levels and inhibition of MPTP opening
Wang *et al*. 20	Rat	Brain	10 min.–48 hrs	60% increase in the number of survived CA1 cells	Improved cerebral blood flow and reduced cytochrome‐C expression
Robin *et al*. 62	Rat	Brain	60 min.–24 hrs	50% decrease in infarct volume	Inhibition of MPTP opening
Liang *et al*. 63	Rat	Brain	2 hrs–24 hrs	44% decrease in infarct volume	Reduced mitochondrial ROS, membrane depolarization, and swelling
Li *et al*. 64	Rat	Intestine	60 min.–60 min	43% decrease in tissue injury based on Chiu index	Antioxidative and anti‐apoptotic effects
Wen *et al*. 21	Rat	Intestine	30 min.–120 min.	31% decrease in tissue injury based on Chiu index	Increased activity of aldose reductase and oxidative defence
Liu *et al*. 65	Rat	Intestine	60 min.–60 min.	25% decrease in tissue injury based on Chiu index	Reduced oxidation injury, neutrophil infiltration, and pro‐inflammatory cytokines
Wen *et al*. 44	Rat	Intestine	60 min.–120 min.	23% decrease in tissue injury based on Chiu index	Reduced activity of JAK/STAT signalling pathway and apoptosis
Tan *et al*. 66	Rat	Kidney	30 min.–7 days	57% decrease in creatinine levels after 3 days, and 20% after 7 days	Increased SDF‐1 expression and modulation of oxidative stress
Zhuang *et al*. 22	Mice	Kidney	26 min.–48 hrs	6% Increase in BUN and 7% creatinine levels *versus* controls	No protective effect of IPostC on renal injury in C57/black mice
Szwarc *et al*. 67	Mice	Kidney	30 min.–8 days	45% decrease in creatinine levels	Reduced acute renal failure in Swiss mice
Knudsen *et al*. 68	Rat	Liver	60 min.–4 hrs, 24 hrs	18% reduction in ALT levels and 8% in AP levels	Reduced necrosis levels
Knudsen *et al*. 17	Rat	Liver	30 min.–30 min.	90% increase in ALT levels, no change in AP and 20% increase in bilirubin level	No protective effect of IPostC and Reduced expression of HIF‐1α mRNA
Xia *et al*. 69	Rat	Lung	40 min.–105 min.	21% reduction in lung injury score	Induced HO‐1 expression
Xu *et al*. 70	Rat	Lung	40 min.–120 min.	36% reduction in lung injury score	Reduced systematic inflammatory response and Induced HO‐1 expression
Cao *et al*. 16	Rat	Lung	30 min.–24 hrs	39% reduction in apoptotic index	Antioxidative, anti‐inflammatory and anti‐apoptotic mechanisms
Song *et al*. 19	Rabbit	Spinal Cord	20 min.–48 hrs	83% reduction in neurologic function score	Up‐regulated activity of endogenous antioxidant enzymes triggered by ROS
Minutoli *et al*. 18	Rat	Testes	60 min.–24 hrs	77% reduction in Lobular coagulative Necrosis	Anti‐apoptotic and anti‐inflammatory mechanisms

IPostC, ischaemic postconditioning; GSK‐3β, glycogen synthase kinase‐3beta; MPTP, mitochondrial permeability transition pore; ROS, reactive oxygen species; JAK/STAT, Janus kinase/signal transducer and activator of transcription; SDF‐1, stromal cell‐derived factor‐1; BUN, blood urea nitrogen; ALT, alanine aminotransferase; AP, alkaline phosphatase; HIF‐1α, hypoxia‐inducible factor 1‐alpha; HO‐1, heme oxygenase 1; CK‐MB, creatine phosphokinase; TnI, troponin I; WMSI, wall motion score index; CTFC, corrected TIMI frame count; MDA, malondialdehyde; SPECT, single−photon emission computed tomography; LVEF, left ventricular ejection fraction; iNOS, inducible nitric oxide synthase; AMI, acute myocardial infarction; PCI, percutaneous coronary intervention.

a The percentages are approximated values according to the main findings of the studies.

## The reasons for the variation of effectiveness of IPostC algorithms in various tissues

**Figure 3:**
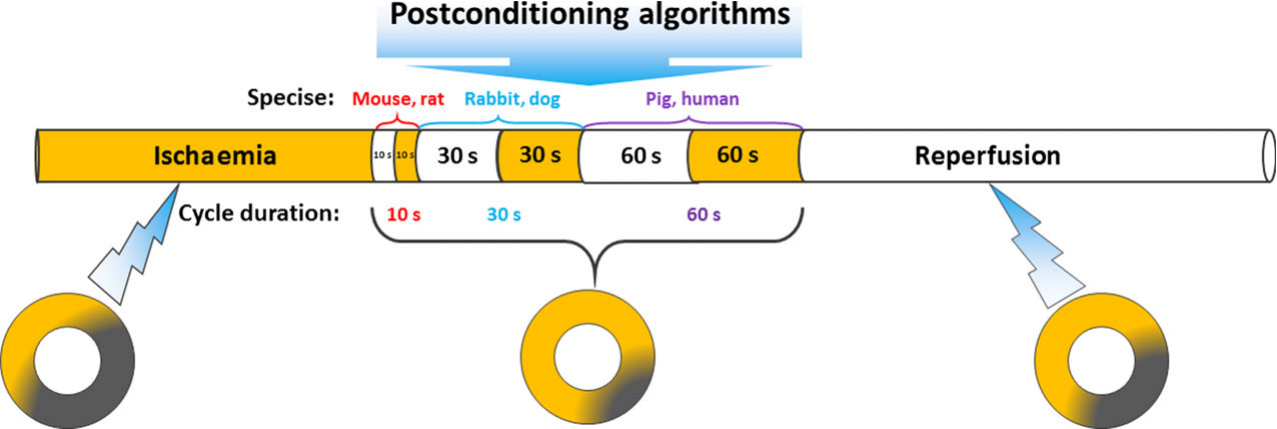
IPostC algorithms, applied at the onset of reperfusion, vary from species to species. IPostC as a simple mechanical intervention during reperfusion has considerable therapeutic aspects in various vital organs undergoing ischaemia and reperfusion injury.

Since the introduction of IPostC, many types of research have been conducted *in vitro* and *in vivo* models of rats, rabbits, mice, dogs, pigs and in humans to confirm the effectiveness of the IPostC protocols. Even though some studies have reported no effect of a given IPostC protocol, research is still going on to figure out its significant effects 37. IPostC effects are undoubtedly affected by its algorithms, including a number of cycles, duration of each occlusion/reperfusion episode and the entire duration of IPostC modality 49. The algorithms with an extra number of cycles or with a duration longer than a critical period abolish the protective effect of IPostC on the myocardium 50. According to the studies in various species, IPostC algorithms may be varied based on animal's heart rate and metabolic rate, so that in small‐body animals like rats and mice which have higher heart rates and metabolic rates, shorter periods (5–10 sec.) of occlusion/reperfusion episodes have demonstrated better effects. Whereas, in larger species such as dogs and pigs, which have lower heart rates, longer duration (30–60 sec.) of episodes works better. In human, typically longer periods (minutes) have been used (Fig. 3). On the other hand, there is still no acceptable reason for this difference and disparity 50, 51. Similarly, an optimized protocol that can be applicable for all species and various tissues has not been identified yet.

Several possible factors contributing to this variation include age, gender, race and species and nutritional conditions of the animals used for study, as well as the duration of ischaemia or other methodological biases. As the ageing complications and related comorbidities are manifested in different forms in different tissues, the effect of similar IPostC algorithm will not be necessarily similar in tissues with different age. With advancing age as well as in the presence of comorbidities, the functions and responsiveness of tissues are declined in a different manner. So, it is obvious that the effect of IPostC strategy and other protective protocols can be affected by the age of the animals selected by each study. The same issue would be true about the gender, race and species or other features of the animals. Next contributor is the ischaemic time. As the tissues differently withstand a certain time of ischaemia (due to different metabolic rate and perfusion levels of tissues as discussed below), whatever the duration of ischaemia rises, the functional recovery of different tissues during reperfusion would be achieved differently. Thus, this issue may negatively or positively affect the outcome of IPostC algorithms.

**Figure 4:**
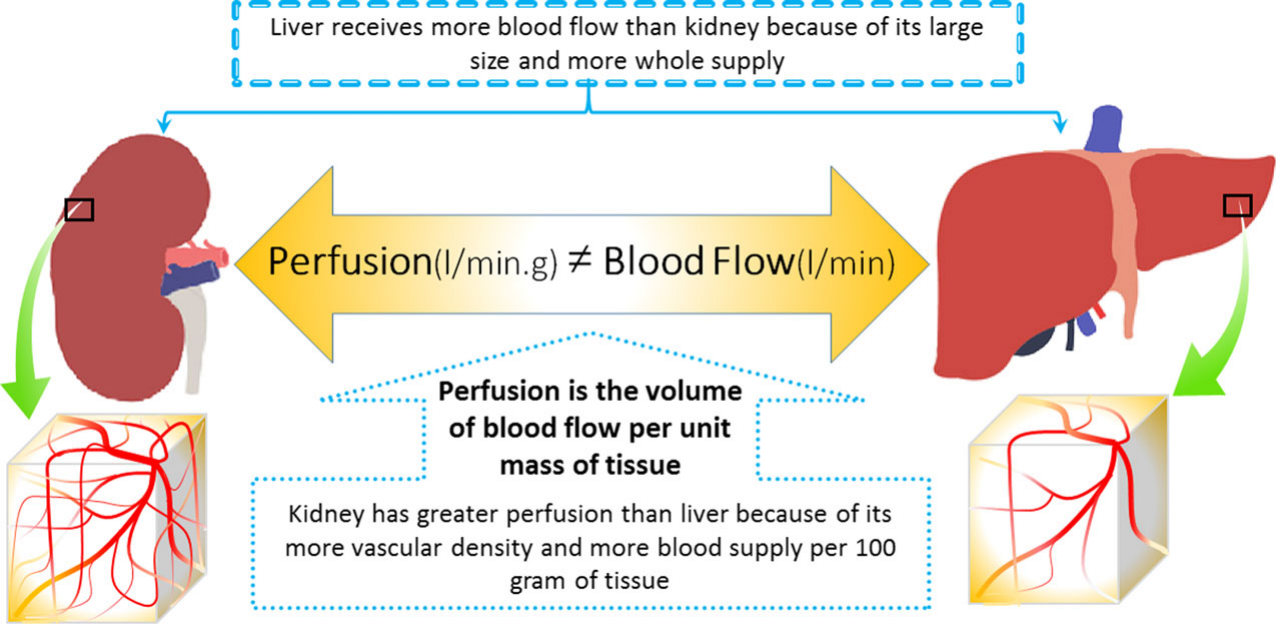
Unequal blood flow and perfusion of tissues.

On the other hand, IPostC algorithms should be designed based on the kind of tissues or organs used in different species, because some features of the tissue such as the nutrients’ requirements, and the volume, and the velocity of blood flow vary from tissue to tissue. In other words, a selected IPostC algorithm should be matched with the tissues’ requirements for nutrients, vascular density and metabolic status, activity levels of intracellular protein kinases and signalling pathways of the tissues and so on. One algorithm may exert very good effects in the protection of one tissue, but it has a moderate effect in another tissue and very weak or even negative impact in another else. This is also true for different species. Thus, in certain tissue (and species), one algorithm may work very better than the others. For example, the heart tissue is extremely sensitive to oxygen, so its IPostC algorithm patterns would be different from those of skeletal muscle which it has compensatory mechanisms during the lack of blood supply such as consumption of oxygen stored in myoglobin. In addition, blood flow (l/min.) and perfusion (l/min. × g of tissue) are two very important variables of each tissue that are associated with this issue. IPostC is extremely dependent upon the perfusion than blood flow *per se*. If these two parameters are compared in liver and kidney (Fig. 4), the liver has greater blood flow but lower perfusion rate than the kidney which has a greater distribution of blood vessels in given mass of the tissue. Thus, if the same algorithm of IPostC has different effects in these tissues, one can attribute this discrepancy to the inequality in perfusion levels of the tissues.

According to the comparison of similar algorithms of IPostC having diverse results in different tissues (Table 2), it can be concluded that selecting a certain IPostC algorithm for all tissues is yet questionable and we cannot easily optimize the same algorithm for any tissue, because of diversity in tissues’ physiological requirements. Therefore, different features of tissues (and species) should be considered when designing an optimal working IPostC algorithm for the protection of those tissues against IR insults.

## Conflict of interest

Authors declare that they have no conflict of interest.
